# Efficacy of a Modified Live Virus Vaccine against Porcine Reproductive and Respiratory Syndrome Virus 1 (PRRSV-1) Administered to 1-Day-Old Piglets in Front of Heterologous PRRSV-1 Challenge

**DOI:** 10.3390/pathogens10101342

**Published:** 2021-10-18

**Authors:** Heinrich Kreutzmann, Sophie Dürlinger, Christian Knecht, Michaela Koch, Marta Cabana, Gerard Torrent, Mònica Balasch, Lucas P. Taylor, Gyula Balka, Wilhelm Gerner, Andrea Ladinig

**Affiliations:** 1 University Clinic for Swine, Department for Farm Animals and Veterinary Public Health, University of Veterinary Medicine Vienna, 1210 Vienna, Austria; heinrich.kreutzmann@vetmeduni.ac.at (H.K.); sophie.duerlinger@vetmeduni.ac.at (S.D.); christian.knecht@vetmeduni.ac.at (C.K.); michaela.koch@vetmeduni.ac.at (M.K.); 2 Zoetis Manufacturing and Research Spain S.L., Ctra. Camprodon s/n Finca La Riba, 17813 Girona, Spain; marta.cabana@zoetis.com (M.C.); gerard.torrent@zoetis.com (G.T.); monica.balasch@zoetis.com (M.B.); 3 Global Development & Operations, Zoetis, Kalamazoo, MI 49007, USA; lucas.p.taylor@zoetis.com; 4 Department of Pathology, University of Veterinary Medicine, 1078 Budapest, Hungary; balka.gyula@univet.hu; 5 Institute of Immunology, Department of Pathobiology, University of Veterinary Medicine Vienna, 1210 Vienna, Austria; wilhelm.gerner@pirbright.ac.uk; 6 The Pirbright Institute, Biotechnology and Biological Sciences Research Council (BBSRC), Woking GU24 0NF, UK

**Keywords:** PRRSV, AUT15-33, vaccination, MLV, 1-day-old pigs, viremia, shedding, cellular immune response, IFN-γ

## Abstract

PRRSV is one of the most important viruses in the global swine industry and is often controlled by the use of modified live virus (MLV) vaccines. This study assessed the impact of a PRRSV-1 MLV vaccine applied to 1-day-old piglets challenged on day 28 of life with a PRRSV-1 field isolate (AUT15-33). Twenty-one piglets were vaccinated within 24 h of birth (T02), whereas 20 piglets were left unvaccinated (T01). Necropsy was performed two weeks post-challenge. Comparing the two groups, T02 piglets showed significantly higher (*p* = 0.017) average daily weight gain. In addition, significantly lower (*p* < 0.0001) PRRSV RNA loads were measured in serum of T02 piglets at all investigated time points. All T01 piglets were viremic and shed virus in nasal swabs, whereas only 71.4% and 38.1% of the T02 group were viremic or shed virus, respectively. Piglets from T02 had significantly higher numbers (*p* < 0.0001) of IFN-γ producing lymphocytes compared to T01. At necropsy, differences in gross and histologic lung lesions were statistically significant (*p* = 0.012 and *p* < 0.0001, respectively) between the two groups. Hence, this MLV vaccine administered to 1-day-old piglets was able to protect piglets against PRRSV infection at weaning.

## 1. Introduction

Thirty years after its discovery, porcine reproductive and respiratory syndrome virus (PRRSV), a small RNA virus belonging to the family *Arteriviridae*, remains one of the most devastating viral pathogens in pig production, both in Europe and worldwide [1,2]. According to the current classification, PRRSV can be divided into two distinct species, PRRSV-1 and PRRSV-2 [3]. Within the individual species, there is a high degree of variability, which has led to a further subdivision into at least three subtypes for PRRSV-1 and nine lineages for PRRSV-2 [4,5].

PRRSV-1 subtype 1 is the dominating subtype among circulating PRRSV isolates in Western, Central and Central Eastern European countries [5,6]. However, in recent years the emergence of highly pathogenic (HP) PRRSV-1 isolates has been observed in Europe, causing reproductive failure in sows and severe clinical signs in pigs of different age groups [7,8,9,10]. One of these HP isolates in Central Europe is AUT15-33, whose virulence has already been phenotypically characterized in case reports and experimental studies [9,11]. In the nursery, an infection with PRRSV causes high economic losses due to increased mortality, increased feed conversion ratio and decreased average daily weight gain [12]. The presence of HP isolates in Europe, resulting in severe clinical signs in nursery and fattening pigs, emphasizes the need for vaccines that reliably provide sufficient protectivity against heterologous PRRSV strains to mitigate the clinical courses after infection.

In Europe, several modified live virus (MLV) vaccines are licensed and are used to minimize the clinical and economic impact of PRRSV infections in nursery and finishing pigs [13,14,15]. Due to the fact that the onset of immunity of vaccinated piglets is delayed and usually detectable around 3–4 weeks after vaccination [16], a gap in protection may occur if vaccination is given late in the suckling period or at weaning. In particular, the potential early circulation of PRRSV in piglets after weaning has led to the development of vaccines with an approval for usage from the first day of life for both PRRSV-1 and PRRSV-2 [17,18].

Various studies have been conducted to characterize the efficacy of early vaccination of suckling piglets against PRRSV-1. It has been shown in an experimental model that maternally derived antibodies do not limit the effect of the Suvaxyn^®^ PRRS MLV vaccine (Zoetis Belgium SA, Ottignies-Louvain-la-Neuve, Belgium) administered to piglets on the first day of life, measured by viral load in serum, nasal and oral shedding after infection with a well-characterized PRRSV-1 field isolate in the 10th week of life [17]. A follow-up study evaluated the ability of piglets to develop a long-lasting cellular and humoral immune response in 1-day-old piglets which were vaccinated and challenged with the same PRRSV field isolate in week 18 of life. It was demonstrated that the immune system of piglets was able to significantly increase interferon gamma (IFN-γ) producing cells after PRRSV-1 MLV vaccination on their first day of life [19]. The acquired cellular immune response, especially the number of antigen-specific IFN-γ secreting cells, is of particular importance for the elimination of the virus [20,21].

The aim of the present study was to determine the level of protective immunity built up after PRRSV-1 vaccination during a four-week suckling period against heterologous challenge. For this purpose, PRRSV-seronegative piglets were vaccinated within the first 24 h of life and exposed to a heterologous HP PRRSV-1 isolate at weaning. Clinical signs, viral load in serum, nasal/oral shedding of the virus and the development of characteristic histopathologic lesions were investigated in vaccinated and non-vaccinated piglets in a two-week period after challenge. To measure the induction of a PRRSV-specific immune response, both an IFN-γ enzyme-linked immunospot (ELISpot) assay, as well as an enzyme-linked immunosorbent assay (ELISA) for PRRSV-specific antibodies were performed.

## 2. Results

### 2.1. Clinical Signs and Body Weight

**Figure 1:**
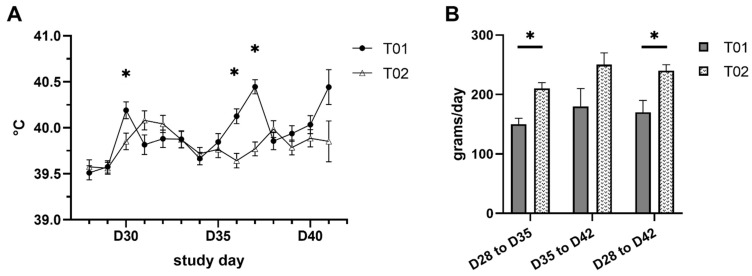
Rectal temperature and average daily weight gain: (**A**) Comparison of least squares means estimates of rectal temperatures between the two treatment groups on the day of challenge (D28) and after challenge until the end of the study (D29–D42). (**B**) Comparison of average least squares means of daily weight gain between the two treatment groups from the day of challenge (D28) until the end of the study (D42). Whisker lines indicate the standard error. An asterisk (*) denotes a significant difference (*p* ≤ 0.05) between vaccinated (T02) and control pigs (T01) at the specific time point/period.

Clinical signs and body weights were measured to monitor the general health condition of the piglets and to detect PRRSV specific symptoms. Twenty-one piglets of the treatment group 2 (T02) were vaccinated on the first day of life, twenty piglets of the treatment group 1 (T01) were sham-inoculated with saline solution. Sneezing was documented most frequently; four piglets from each group showed this sign. After challenge, in the T01 group eleven animals showed depression and seven showed respiratory distress. In contrast, in the vaccinated group four animals showed depression and one showed respiratory distress. The incidence of depression and respiratory distress in the T01 group was statistically higher than that observed in the T02 group (*p* = 0.027 and *p* = 0.042, respectively). Statistically significant differences (*p* ≤ 0.05) between the two treatment groups in rectal temperature were observed on D30, D36 and D37. The course of rectal temperature in both treatment groups is displayed in Figure 1A. Two piglets from T01 showed a severely compromised clinical condition (bad general condition, depression, respiratory distress, cough and fever; the total score of ≥9 was reached) and had to be euthanized due to welfare reasons on D38 and D41.

The comparison of least squares means (LSM) of body weight showed no significant differences between T01 and T02 groups on the day of challenge (D28) or post-challenge (D35 and D41/42). Nevertheless, mean average daily weight gain (ADG) from day of challenge until the end of the study was 170 g ± 20 g and 240 g ± 10 g for T01 and T02 piglets, respectively (*p* = 0.017). Results of ADG are summarized in Figure 1B.

### 2.2. Gross and Histologic Lung Lesions

**Figure 2:**
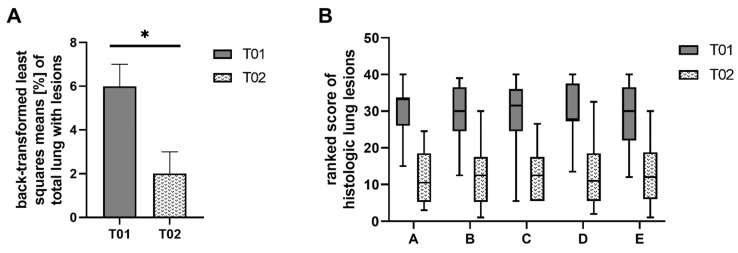
Macroscopic and histologic lung lesion score: (**A**) Comparison of macroscopic lung lesions between the two treatment groups at necropsy. The percentage of consolidation for each lobe was scored and a total score of the seven lung lobes was calculated using the following formula: Percentage of total lung with lesions = (0.10 × left cranial) + (0.10 × left middle) + (0.25 × left caudal) + (0.10 × right cranial) + (0.10 × right middle) + (0.25 × right caudal) + (0.10 × accessory). The arcsine square root transformation was applied prior to statistical analysis. For visualization, least squares means were back-transformed. The whiskers indicate the standard error, the asterisk (*) denotes a significant difference (*p* ≤ 0.05) between vaccinated (T02) and control pigs (T01). (**B**) Comparison of five histologic lung lesions between vaccinated (T02) and control (T01) pigs (A: Intra-alveolar accumulation of inflammatory cells; B: Perivascular accumulation of inflammatory cells; C: Necrotic debris; D: Pneumocytic hypertrophy and hyperplasia; E: Septal infiltration with mononuclear cells). The lesions were scored according to their severity from 0 (no lesions) to 3 (severe lesions) and extension from 0 (absent) to 3 (diffuse) within each of the seven lung lobes. The maximum achievable score for each lesion per animal was 42. To compare the different histologic lesions with each other, the scores for each lesion were ranked. Box and whisker: min-max.

**Figure 3:**
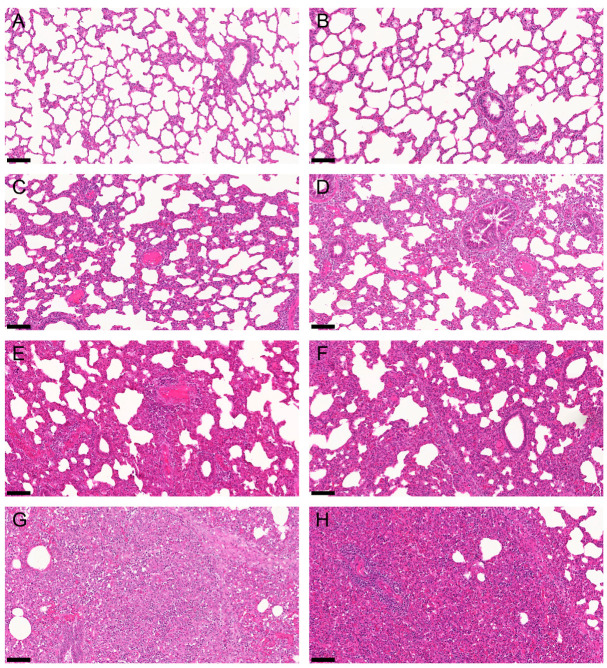
Representative histological slides of non-affected (**A**,**B**), mildly affected (**C**,**D**), moderately affected (**E**,**F**), and severely affected (**G**,**H**) lung lobes. Each lung lobe was scored for severity from 0 (no lesions) to 3 (severe lesions) and extension from 0 (absent) to 3 (diffuse) in five different parameters: intra-alveolar accumulation of inflammatory cells, perivascular accumulation of inflammatory cells, necrotic debris, pneumocytic hypertrophy and hyperplasia, and septal infiltration with mononuclear cells. The maximum achievable total score in a given lobe was 30. (**A**) Right caudal lobe of vaccinated piglet #49, total score 0/30. (**B**) Right caudal lobe of vaccinated piglet #68, total score 0/30. (**C**) Right caudal lobe of vaccinated piglet #32, total score 7/30. (**D**) Left cranial lobe of vaccinated piglet #49, total score 8/30. (**E**) Left cranial lobe of non-vaccinated piglet #66, total score 18/30. (**F**) Left cranial lobe of non-vaccinated piglet #25, total score 15/30. (**G**) Left caudal lobe of non-vaccinated piglet #21, total score 27/30. (**H**) Right cranial lobe of non-vaccinated piglet #48, total score 25/30. H&E staining was used. All slides are at 15 × magnification, bars at the left bottom indicate 100 μm.

Comparison between the two treatment groups showed significant differences in the percentage of total lung with macroscopic lesions. These lesions were significantly reduced (*p* = 0.012) in the T02 group and ranged between 0% and 22% in T01 and 0% and 11% in T02 (Figure 2A). Moreover, the severity and extension of all five individual histologic lung lesions were significantly reduced (*p* < 0.0001) in vaccinated piglets (T02) compared to non-vaccinated piglets of T01. Boxplots of the ranked scores are shown in Figure 2B. Additionally, LSM differences between the two treatment groups, degrees of freedom (df), T-values and *p*-values are displayed in Table 1. Comparing the five histopathologic lesions between the two groups, the ranked LSM in group T01 was 16.0 to 18.4 points higher, with 36 df for perivascular accumulation of inflammatory cells and 38 df for the other four histopathologic lesions. Representative histologic slides of non-affected, mildly, moderately, and severely affected lung lobes are shown in Figure 3.

### 2.3. Viremia and Viral Shedding

**Figure 4:**
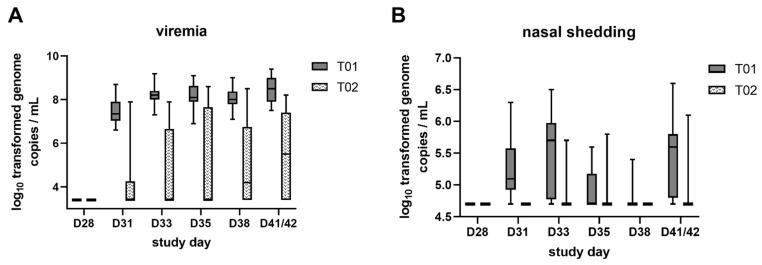
Comparison of viremia (**A**) and nasal shedding (**B**) between vaccinated (T02) and control pigs (T01) at the day of challenge and at five time points after challenge using a PRRSV AUT15-33 specific RTq-PCR (log_10_ transformed data). Results below the cut-off were raised to the limit of detection (3.4 log_10_ genome copies/mL for viremia and 4.7 log_10_ genome copies/mL for nasal swabs). Box and whisker: min-max.

Viremia and viral shedding were investigated to prove the PRRSV negative status of piglets before experimental infection and to monitor virus replication and excretion after challenge. All piglets were negative in the PRRSV AUT15-33 specific reverse transcription quantitative PCR (RTq-PCR) in serum before vaccination (D0) and before challenge (D28). By D31, all pigs from T01 developed viremia and remained AUT15-33 RTq-PCR positive throughout the monitoring period. In contrast, 71.4% of the piglets in T02 were PRRSV PCR positive at one or more time points post-challenge. With the exception of one T02 piglet, the piglets that were positive once remained positive until the end of the study. The log_10_ transformed genome copies/mL serum per treatment group are shown in Figure 4A. The viral load detected in serum was significantly higher (*p* < 0.0001) in T01 compared to T02 group piglets at all monitored time points after challenge. Comparing the mean portion of days for a piglet to be viremic, the T02 group had fewer viremic days (30.6%) compared to the T01 group (83.2%). Additionally, the mean area under the curve (AUC) of log_10_ transformed viremia data up to D41/42 was significantly higher (*p* < 0.0001) in T01 piglets compared to T02 piglets with LSM log_10_ values of 9.4 ± 0.3 and of 6.8 ± 0.3, respectively.

Shedding was monitored by the detection of PRRSV AUT15-33 by RTq-PCR in nasal and oral swab samples. Shedding was not detected before vaccination (D0) and before challenge (D28). After challenge, all of the T01 piglets shed virus in nasal swabs, whereas less than half of the T02 piglets shed virus (38.1%). In oral swabs, 50% of T01 piglets and 9.5% of T02 piglets were classified positive. In T02, the portion of days with nasal shedding was 2.9% and with oral shedding 0.2%, while in T01 it was 43.4% and 4.9%, respectively. In contrast to viremia, discontinuous nasal shedding of virus was observed among the piglets of both treatment groups. The log_10_ transformed genome copies/mL in nasal swabs per treatment group are displayed in Figure 4B. The viral load LSM detected in nasal swabs were significantly higher in T01 compared to T02 group piglets on D31, D33 and D41/42. No significant differences were found for oral swabs. The mean AUC of log_10_ transformed shedding data up to D41/42 was significantly higher (*p* < 0.0001) in T01 piglets compared to T02 piglets for nasal shedding but not significant for oral shedding. LSM differences between the two treatment groups, standard error (SE), df and *p*-values for both viremia and nasal shedding are displayed in Table 2.

### 2.4. IFN-γ Producing Peripheral Blood Lymphocytes and PRRSV Nucleoprotein Antibodies

The frequencies of IFN-γ secreting cells were investigated within 3 × 10^5^ peripheral blood mononuclear cells (PBMC) upon re-stimulation with PRRSV antigen by the use of an IFN-γ ELISpot assay.

**Figure 5:**
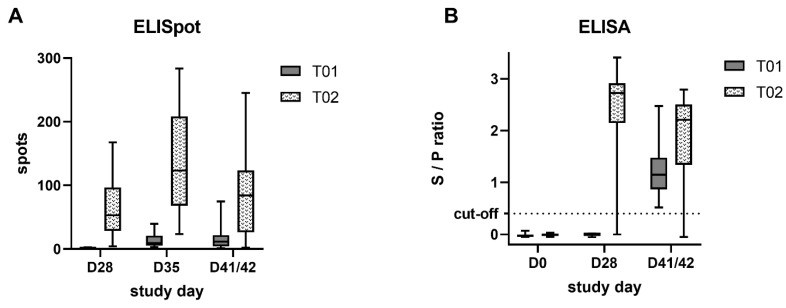
IFN-γ ELISpot and ELISA results: (**A**) comparison of ELISpot data after PRRSV re-stimulation of 3 × 10^5^ PBMC between vaccinated (T02) and control pigs (T01) at challenge (D28), seven days later (D35) and at necropsy (D41/42). (**B**) Comparison of ELISA S/P ratios between vaccinated (T02) and control pigs (T01) at vaccination (D0), at challenge (D28), and at necropsy (D41/42). The ELISA was considered positive at an S/P ratio of ≥0.4. Box and whisker: min-max.

Piglets from T02 had significantly higher (*p* < 0.0001) IFN-γ producing cells compared to the control group at all monitored time points. Results of IFN-γ ELISpot are shown in Figure 5A. The overall highest number (LSM) of IFN-γ producing cells was observed in the T02 group seven days post-challenge (D35). Numbers of detected spots ranged from 21 to 281 as indicated by the whiskers. In the T01 group, it ranged from 1 to 38 spots on the same study day. For D41/42 it ranged from 0 to 243 spots and from 0 to 73 spots in the T02 and T01 group, respectively. LSM differences between the two treatment groups, SE, df, and *p*-values are displayed in Table 3.

In the IDEXX PRRS X3^®^ ELISA (IDEXX PRRS X3^®^ Ab Test, IDEXX Europe B.V., Hoofddorp, The Netherlands), all pigs were negative prior to vaccination (D0). Prior to the challenge (D28), all T01 piglets were negative, whereas all but one of the T02 piglets (piglet 14) had seroconverted (S/P ratio LSM 0.00 ± 0.00 vs. 2.44 ± 0.17). At the time of necropsy, all but one of the piglets (T02, piglet #9) had S/P ratios above the cut-off, although the S/P ratio was significantly higher (*p* = 0.002) in the T02 group (LSM 1.23 ± 0.11 vs. 1.89 ± 0.17). ELISA S/P ratios of the two treatment groups at the different time points are displayed in Figure 5B. LSM differences between the two treatment groups, their SE, df, and p-values for D28 and D41/42 are indicated in Table 4.

### 2.5. Correlation of Viremia and IFN-γ Producing Peripheral Blood Lymphocytes

**Figure 6:**
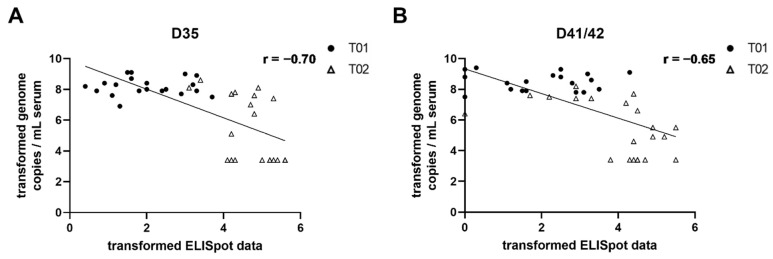
Correlation of viremia and IFN-γ ELISpot data from all piglets on D35 (**A**) and D41/42 (**B**). The Pearson correlation coefficient (r) is indicated and the simple linear regression line is plotted. The piglets of the two treatment groups (T01 = control; T02 = vaccinated) are indicated by different symbols (filled circles [•]: T01 piglets; triangles [∆]: T02 piglets). An appropriate logarithm transformation was conducted prior to statistical analysis and visualization.

A correlation of log transformed viremia and ELISpot data from all piglets on D35 and D41/42 was calculated. Overall, higher numbers of IFN-γ producing lymphocytes resulted in lower amounts of virus in the blood (Figure 6). The Pearson correlation coefficient (r) showed a significant negative correlation (*p* < 0.0001) at both time points with r = −0.70 on D35 and r = −0.65 at necropsy.

## 3. Discussion

Weaning-age pigs and growing pigs are of particular epidemiological relevance because of their susceptibility to PRRSV when the virus is circulating endemically in the herd [22]. The importance of mitigating the effects of PRRSV infection in growing pigs is reflected in an economic evaluation that divides the overall economic losses due to PRRSV in the United States between breeding herds and growing pig herds. More than 50% of the economic losses due to PRRSV are incurred by growing pigs [1]. It has been described that PRRSV can cause cyclic waves of clinical signs and mortality in nursery pigs of endemically infected herds even without reproductive failure in sows [23]. In addition, infection dynamics in the nursery differ between endemically infected farms [24]. Considering that in endemically infected herds individual litters become infected in utero or during the suckling period [25], breeding herd stabilization is necessary to produce non-viremic piglets [26]. However, commingling of susceptible piglets with infected piglets occurs at weaning when piglets from different litters are mixed and furthermore virus is circulating between the different age groups in the nursery. Thus, the possibility of PRRSV infection among the piglets after weaning is increased. To mimic a potential early infection, piglets in this study were challenged at weaning on day 28 of life. For determination of the protective effect of the vaccine, half of the piglets were vaccinated within the first 24 h of life.

The chosen PRRSV isolate has been shown to be highly virulent in previous infection trials [11]. This finding was also corroborated in the current study, as indicated by PRRSV-specific clinical signs and high viral loads in serum and tissues, especially in the non-vaccinated infected piglets. A substantial increase in viral load LSM was observed at the first investigation time point three days post-challenge (7.4 ± 0.1 log_10_ genome copies/mL serum in T01). Overall, highest viral loads were found in serum five days post-challenge and at the day of necropsy (8.2 ± 0.1 and 8.5 ± 0.1 log_10_ genome copies/mL serum in T01). This corresponds to an absolute viral load of 1.7 × 10^8^ and 3.4 × 10^8^ genome copies/mL serum, respectively. Thereby, this finding is in line with the observation of an AUT15-33 outbreak in the field, where genome copy numbers ranged between 1.4 × 10^8^ and 4.3 × 10^9^ in serum of acutely affected nursery piglets [9]. Canelli et al. described similar results in an experimental trial with a highly pathogenic Italian PRRSV-1 subtype 1 isolate PRRSV-1_PR40/2014 (PR40). This strain caused severe clinical signs in growing pigs in the field and thereafter was isolated on primary porcine alveolar macrophages (PAM). Seven 5-week-old piglets from a PRRSV-free herd were intranasally infected with PR40 at dose of 10^5^ tissue culture infectious dose 50% (TCID_50_) per pig. Viral load peaks were reached 7 days post-challenge and ranged approximately from 8.4 to 10.2 log_10_ genome copy numbers/mL serum [10]. In a study performed in the US by Johnson et al., the viremia levels after infection with eight PRRSV-2 isolates (five virulent field isolates and three attenuated PRRSV isolates) were compared. Virulent PRRSV isolates exhibited longer and more elevated levels of viremia and caused more severe clinical signs in a respiratory disease model. In contrast to the animals infected with the attenuated isolates, a substantial increase in average viral load on day 1 post-challenge was detected in piglets infected with virulent strains. In these groups viral load peaked above 8 log_10_ genome copy numbers/mL serum between day 7 and 15 post-challenge. In contrast, viremia levels in the piglets infected with the attenuated strains peaked on different days of the study (3, 28, and 35 days post-challenge) and also showed a lower level (4.3 to 6.9 log_10_ genome copy numbers/mL serum). The authors indicated that the growth rate of PRRSV in pigs is a phenotypic characteristic of the particular isolate with highly virulent strains replicating at a much higher rate [27].

In general, commercial MLV vaccines have been effective at providing protection against homologous strains, whereas different levels of cross-protection after challenge with heterologous PRRSV strains can be observed [28,29,30]. Regarding highly pathogenic PRRSV-1 isolates, the ability of MLV vaccines to provide partial protection has been demonstrated in several experimental studies [13,14,31]. In these studies, however, the animals were vaccinated after weaning and challenged experimentally at the earliest four weeks after vaccination. These time points suggest an infection in the middle or towards the end of the nursery period. The present experiment proves that the current MLV vaccine is also able to significantly reduce pronounced clinical signs such as depression and respiratory distress when administered on the first day of life followed by challenge at weaning. Non-vaccinated piglets showed a typical biphasic rise in rectal temperature on day 2 and on days 7–9 after PRRSV infection. This phenomenon can often be observed in experimental studies [32,33] and might be due to initial virus replication and viral load peak, respectively [34]. Remarkably, the average daily weight gain of vaccinated piglets was significantly higher in the 14-day-period after infection. Vaccinated piglets had on average 70 g higher weight gain, which also indicates the positive economic impact of the vaccination.

To assess typical PRRSV-induced lung lesions, an early necropsy time point two weeks post-challenge was chosen. PRRSV-specific lung lesions are likely to decrease significantly after the acute phase of infection [35]. In the acute phase, PRRSV infection in growing pigs mainly affects the lung, especially the alveolar macrophages, which are primary target cells of PRRSV [36]. Additionally, apoptosis of bystander cells in the lung parenchyma modulated by cytokines and chemokines released by virus-infected cells has been described [37,38]. In the current setup, all five investigated lesions were less pronounced in vaccinated piglets, indicating that the vaccine can substantially reduce PRRSV-specific lung lesions. The virus-induced impairment of the lung tissue and local defense mechanisms favor the damaging effect of opportunistic bacterial infections. Due to predisposing factors on farms, it can therefore be assumed that PRRSV infections take a substantially more severe course in field cases compared to experimental studies [39]. After an AUT15-33 outbreak in the field, swollen joints, stomach ulcers, pericarditis, and a catarrhal to purulent bronchopneumonia were observed, probably due to a co-infection with *Staphylococcus hyicus*. A basic immunization of all pigs on site at intervals of four weeks with a PRRS MLV vaccine was implemented directly after PRRSV detection in the specific herd. However, losses in the nursery were limited to mainly one age group and clinical signs declined in the nursery after the implementation of the vaccination protocol [9].

In addition, the use of the Suvaxyn^®^ PRRS MLV vaccine has already been described in the field. In a PRRSV-positive and unstable farm, 636 piglets were randomly allocated into two treatment groups: a control group and a vaccinated group. The vaccine was administered at 1–4 days of age. Vaccinated pigs showed shorter viremia, a lower maximum number of PRRSV PCR positive pigs, lower levels of wild-type virus shedding and a higher ADG [40].

PRRSV oral shedding has been described in literature starting three days after infection and is considered as a possible route of virus transmission [17,41]. In the present experiment it could only be detected in 10/20 piglets of the T01 group and in 2/21 piglets of the T02 group. The level and duration of viral shedding is dependent on the virus isolate [42]. For the present experiment, it has to be kept in mind that RTq-PCR values below the cut-off were raised to the respective limit of detection as an additional statistical safeguard. However, despite this additional statistical confidence, significant differences in viremia and nasal shedding were detected in this experiment, confirming a positive effect of vaccinating 1-day-old piglets against PRRSV in terms of reducing the risk of viral shedding and transmission. Additionally, it has to be considered that vaccinated and non-vaccinated piglets were commingled after weaning in order to control for the effects of location/room. Due to PRRSV shedding especially by non-vaccinated piglets, it can be assumed that all piglets have been exposed to higher amounts of virus after challenge. Therefore, it is even more important to note that 6 out of 21 vaccinated piglets did not develop any measurable viremia.

One key aspect of this study was to investigate the induction of a PRRSV-specific immune response. ELISA results demonstrated that both vaccination and infection induced antibody formation. The S/P ratio in the vaccinated group was significantly higher compared to the non-vaccinated group, indicating that a humoral immune response was elicited. In addition, the S/P ratio in the vaccinated group at D28 was comparable to that of animals measured five weeks after vaccination against PRRSV at 4 weeks of age with a PRRS-MLV [14,43]. Nonetheless, the protective effect of those antibodies is not sufficient. It is described that early non-neutralizing antibodies do not provide sufficient protection against infection with PRRSV, neither in vitro nor in vivo [44,45]. In in vitro experiments it has also been described that certain monoclonal non-neutralizing antibodies against the N and GP5 protein of the virus can induce an antibody-dependent enhancement (ADE) of virus replication in PAM cultures [46]. However, the biological significance of ADE in vivo remains to be investigated. Various studies have already been conducted to characterize the efficacy of early vaccination of suckling piglets against PRRSV-1, with differing results [17,19,47,48]. Particular attention has been paid to maternally-derived antibodies (MDA) and a potential interference with the development of anti-PRRSV immunity in piglets after vaccination. In a trial performed in France, a PRRSV-1 MLV vaccine other than the one applied in this trial was used to vaccinate 3-week old pigs twice at an interval of one week. In addition, a group of piglets was left unvaccinated. Sows were vaccinated beforehand with the same MLV strain on a regular basis. Piglets were assigned to different treatment groups according to their specific level of neutralizing antibodies targeting the vaccine strain. Challenge with a heterologous PRRSV-1 field strain was performed five weeks after the first vaccination of the piglets. A reduction of viremia, compared to non-vaccinated infected piglets, could only be observed in piglets with lower levels of neutralizing antibodies [48]. For the vaccine used in this experiment, a slightly different setup was used in a previous study [17]. Six seronegative sows were purchased from a PRRSV naïve farm and vaccinated once with Suvaxyn^®^ PRRS MLV at 45 days of gestation. After farrowing, piglets were randomly assigned to two groups. The vaccinated group received a Suvaxyn^®^ PRRS MLV vaccination on the first day of life and the control group was left unvaccinated. In this previous study, 25 of 34 piglets had measurable neutralizing antibody levels against the vaccine strain upon vaccination. Experimental infection with PRRSV-1 isolate Olot/91 took place in week 10 of life, resulting in a significant reduction of viremia in vaccinated animals at all measured time points after challenge compared to non-vaccinated animals [17]. No statement on MDA can be made in the current study, since the sows were PRRSV negative.

In general, immune cells of the adaptive immune system are present at birth, but the absolute number of T-helper cells and cytotoxic T cells in blood are lower compared to older pigs, and most of the T-helper cells are negative for CD8α, indicating a naïve phenotype [49]. IFN-γ has antiviral and pro-inflammatory effects [50] and is produced mainly by natural killer (NK) cells, cytotoxic T cells and Th1 cells, the latter being a subgroup of T-helper cells. The occurrence of PRRSV-specific IFN-γ-producing cells within PBMC was measured by an ELISpot assay [51,52]. As it could be expected for PRRSV-naïve animals, the PBMC of the T01 piglets contained hardly any IFN-γ producing cells on the day of challenge following re-stimulation with PRRSV antigen. In contrast, the PBMC isolated from the T02 group included significantly more IFN-γ producing cells, suggesting an effective priming of PRRSV-specific T cells by the vaccine, despite the young age of the animals. This is comparable to a previous study on the use of this vaccine in 1-day-old piglets; however, direct comparison is not possible due to different methodologies of the laboratories [19]. In our study, an increase in IFN-γ-producing cells was observed in both groups after challenge, but this was significantly higher in the vaccinated group at both time points after challenge. In experimental studies, IFN-γ was able to inhibit PRRSV replication in the simian MARC-145 cell line [53]. Additionally, in in vivo studies, clinical protection was associated with a marked production of virus-specific IFN-γ-producing cells [28,54]. Thus, the increase of IFN-γ-producing cells indicate that 1-day-old piglets are able to establish a measurable, resilient PRRSV-specific cellular immune response within four weeks after vaccination on the first day of life.

Finally, using Pearson Correlation, this study also showed that the number of IFN-γ-producing cells is negatively correlated with the PRRSV AUT15-33 genome equivalents in the blood. This finding confirms that IFN-γ producing cells contribute to the reduction of PRRSV load and emphasizes the relevance of Th1-associated immune measurements in the evaluation of the efficacy of a vaccine against PRRSV.

## 4. Materials and Methods

### 4.1. Animals and Study Design

Four clinically healthy breeding gilts were purchased from a specialized gilt producer (PIC Deutschland GmbH) and housed in a piglet-producing farm in Lower Austria unsuspicious for PRRSV based on routine serological monitoring. Four weeks before farrowing, gilts were transported to the facilities of the University Clinic for Swine, University of Veterinary Medicine, Vienna. One week prior to farrowing, gilts were randomly allocated to four individual rooms with separate air space within a biosafety level 2 (BSL-2) isolation unit. After farrowing, piglets were ear-tagged, weighed and samples were collected. A random treatment allocation plan was provided by the biometrician and piglets were cross-fostered across the gilts. Piglets in two rooms were vaccinated within 24 h after birth (T02 group), piglets in the other two rooms were sham-treated intramuscularly with 2 mL of saline diluent (T01 group). The day of vaccination was considered as D0. Forty-one piglets fulfilled the inclusion criteria, with 20 and 21 piglets in T01 and T02, respectively. Weaning was performed by removal of the sows at D28. After weaning, vaccinated and non-vaccinated piglets were commingled onto the four rooms such that actual littermates were put back together, so that vaccinated and non-vaccinated piglets were kept in the same room. The same day, all piglets in T01 and T02 were challenged with a PRRSV-1 field isolate (see below). All animals had free access to water and were fed ad libitum with a commercial diet. Piglets were followed and samples were collected for two weeks after challenge before necropsy was performed.

### 4.2. Vaccine and Challenge Virus

Vaccination of T02 piglets was performed on the first day of life intramuscularly with 2 mL of a commercial MLV vaccine, Suvaxyn^®^ PRRS MLV (vaccine batch PDLLYO-18-001, Zoetis Belgium SA, Ottignies-Louvain-la-Neuve, Belgium). The vaccine is based on the PRRSV-1 subtype 1 strain 96V198c1. On the day of challenge (D28), all piglets in T01 and T02 were intranasally infected with the PRRSV-1 subtype 1 strain AUT15-33 (Gen-Bank accession number MT000052.1) at a concentration of 1 × 10^5^ TCID_50_/2mL (1 mL administered per nostril). The isolate was propagated on PAM at the Institute of Virology of the University of Veterinary Medicine, Vienna.

### 4.3. Clinical Examination and Necropsy

Only clinically healthy, PRRSV-negative (surveyed by means of RTq-PCR and serological testing) piglets were enrolled in the study. After challenge, piglets were monitored daily for rectal temperature and clinical signs. Piglets were scored for general condition, depression, sneezing/coughing, and respiratory distress. If no deviations from the normal status were observed, the score 0 was assigned; abnormalities were classified in ascending order with a score of 1 to 3. Hyperthermia (≥40.5 °C) was generally given a score of 2. Other conditions were scored individually, nasal discharge and mild digestive disorders were scored with 1, vomiting and lameness was given a score of 2. The resulting total score (sum of individual scores per piglet) was used for statistical evaluation and as end point criteria. A piglet was euthanized for welfare reasons, if a total score ≥9 was achieved or a severe clinical sign (individual clinical sign score = 3) was observed or total scores between 6 and 8 were diagnosed for four consecutive days. Additionally, body weight was assessed on D28, D35 and D41/42.

After euthanasia, pigs were necropsied and the presence and severity of gross lung lesions were assessed using the scoring system previously described by Straw et al. [55]. Briefly, each lung lobe (left cranial, left middle, left caudal, right cranial, right middle, right caudal and accessory) was scored and recorded as percentage of lobe with lesions (0% to 100%). A total weighted lung score was calculated for each lung, taking into account the differently sized lung lobes (weighting factor for caudal lobes and for the other lobes 0.25 and 0.10, respectively).

Additionally, five different histologic lung lesions were evaluated: (1) pneumocytic hypertrophy and hyperplasia, (2) septal infiltration with mononuclear cells, (3) necrotic debris, as well as (4) intra-alveolar and (5) perivascular accumulation of inflammatory cells. Samples were fixed in 10% neutral buffered formaldehyde for 24 h before being embedded in paraffin wax and stained with hematoxylin and eosin (H&E). Each lesion was scored in all seven lung lobes per piglet according to severity from 0 (no lesions) to 3 (severe lesions) and extension from 0 (absent) to 3 (diffuse) by a blinded investigator. The maximum achievable score for each lesion was 42 and the maximum total lung lesion score was 210 [35]. The slides were scanned with a Pannoramic Midi slide scanner instrument (3DHistech, Budapest, Hungary), the digital slides were analyzed, and the representative pictures were taken with the SlideViewer software (3DHistech).

### 4.4. RTq-PCR Analysis

Viremia (PRRSV load in serum) and shedding (PRRSV load in swab material) was measured on D0, D28, every 2–3 days after challenge, and at necropsy. A PRRSV-1 AUT15-33 specific RTq-PCR was performed by use of a Taqman assay (forward primer: AUT15-33F 5′-GCACCATCTCACACAAAC-3′; reverse primer: AUT15-33R 5′-CAACTCCTGCGCCTTGAT-3′; probe: AUT15-33S FAM 5′-CCTCTGCTTGCAGTCGATACAGAC 3′ TAMRA). The RTq-PCR was conducted in a thermocycler (7500 Real-Time PCR System, Applied Biosystems, Thermo Fisher Scientific Inc., Waltham, MA, USA). Cut-off values for considering samples as virus positive were defined as ≥2500 genome copies/mL serum, ≥50,000 and ≥5000 genome copies/mL for nasal and oral swabs, respectively. These values were validated according to the Limit of Detection (LoD) and Limit of Quantitation (LoQ) of the used PCR protocol in different matrices. Before statistical evaluation, all values below the cut-off were raised to the respective cut-off value. In addition, the AUC was calculated for each piglet.

### 4.5. Serology

PRRSV-specific antibodies (Abs) targeting the nucleocapsid (N) protein were measured in serum samples from piglets collected before vaccination (D0), just before challenge (D28) and at the end of the study (D41/42) using the IDEXX PRRS X3^®^ ELISA according to the manufacturer’s instructions (IDEXX PRRS X3^®^ Ab Test, IDEXX Europe B.V., Hoofddorp, The Netherlands). Samples with an S/P ratio equal to or greater than 0.4 were considered positive.

### 4.6. PRRSV-Specific IFN-γ ELISpot

IFN-γ producing lymphocytes were investigated on D28, D35 and D41/42. Prior to the analysis, PBMC were isolated from heparinized blood samples by means of a density gradient centrifugation and a lymphocyte separation medium (Pancoll human, density 1.077 g/mL, PAN-Biotech, Aidenbach, Germany; 30 min at 920× g). Isolated PBMC were analyzed for IFN-γ production by use of an ELISpot assay as previously described [52]. In summary, 96-well microtiter plates with a polyvinylidene fluoride membrane were coated with a monoclonal mouse anti-swine antibody specific to porcine IFN-γ (clone pIFNγ-I, Mabtech AB, Nacka Strand, Sweden). At the day of PBMC isolation, membrane of the plates was blocked for one hour with cell culture medium (RPMI 1640 (PAN-Biotech) supplemented with 10% heat inactivated fetal calf serum (Sigma, Schnelldorf, Germany), 100 IU/mL penicillin and 0.1 mg/mL streptomycin (PAN-Biotech)). Freshly isolated PBMC were resuspended in cell culture medium and cell stimuli and cell suspension were added to the wells. For re stimulation of cells, Suvaxyn^®^ PRRS MLV was used at a multiplicity of infection (moi) = 0.1 and Concanavalin A (positive control) at a concentration of 3 µg/mL. Cells without stimulus (medium only) were tested as negative control. All samples were analyzed at least in duplicates. After incubation at 37 °C and 5% CO_2_ for 24 h, PBMC were removed. Then the plates were incubated for one hour with an IFN-γ specific biotinylated detection antibody (clone P2C11, Mabtech), washed and incubated with streptavidin alkaline phosphatase (Sigma) for one hour. After a washing step, BCIP/NBT (5-bromo-4-chloro-3-indolyl phosphate/nitro blue tetrazolium) substrate (Sigma) was added for 5 min. Spots were analyzed in a camera-based automated counting system (AID^®^ ELISpot reader, Advanced Imaging Devices GmbH, Straßberg, Germany). For statistical analysis, spots of the negative control (spots detected after culture in cell culture medium) were subtracted from the spots after PRRSV re-stimulation.

### 4.7. Statistical Analysis

The piglet was the experimental unit in the challenge phase. The respective gilt was the block using a generalized randomized block design. Frequency distributions of clinical signs following challenge were calculated separately for each treatment and time point. Frequency distributions of piglets ever showing the respective clinical sign were calculated for each treatment. Results were analyzed with a general linear mixed model, logit link and treatment as a fixed and block as a random effect. Pairwise treatment comparisons (*t* test) were made if the treatment was significant. Prior to statistical analysis, RTq-PCR data was transformed using a logarithm base 10 transformation. Rectal temperatures, body weight, RTq-PCR data, ELISA S/P ratios and IFN-γ producing cells were analyzed using a general linear repeated measures mixed model with fixed effects: treatment, time point, and treatment and time point interaction. Random effects were block and piglet within block by treatment. In addition, pairwise treatment comparisons were made for each time point if the treatment or treatment by time point interaction effect was significant. Treatment LSM, SE, the minimum and maximum, and 95% confidence intervals (CI) were calculated for each time point. ADG was estimated using parameter-based model estimates. Comparisons were made after testing for a significant treatment and treatment by time point interaction. For RTq-PCR data, the portion of days with viremia and nasal/oral shedding was calculated and it was determined whether a piglet was ever viremic or ever shed virus nasally and orally. For the analysis of antibody responses, frequency distributions of the positive/negative results were calculated for each treatment at each time point. In addition, it was determined for each piglet whether it seroconverted (≥0.4 S/P ratio) during the study.

The AUC of viremia/shedding data was analyzed using a general linear mixed model with treatment as fixed effect and block as random effect. In accordance with the other statistical analyses, pairwise comparisons were made between the treatment groups if the treatment was significant. Additionally, LSM, SE and 95% CI were calculated as well as the minimums and maximums for each treatment. The percentage of total lung with macroscopic lesions was transformed using the arcsine square root transformation. The scores for each histologic lung lesion were ranked and the ranks as well as the transformed macroscopic lung lesions were analyzed in the same manner as AUC data. For comparison of viremia and CMI, a correlation of corresponding log transformed RTq-PCR and IFN-γ ELISpot results was computed using a Pearson correlation coefficient.

Only data from D28 onwards when piglets were commingled was analyzed. Data summaries and analyses were performed with SAS/STAT (Version 9.4 or higher, SAS Institute, Cary, NC, USA). For visualization, GraphPad Prism 9.0 (GraphPad Software, San Diego, CA, USA) was used.

## 5. Conclusions

This experiment confirmed the phenotypic virulence of the PRRSV-1 field isolate AUT15-33. Under the conditions of this trial, piglets are already vaccinable on the first day of life. An effective immunity could be established, which protected the piglets against early PRRSV-1 AUT15-33 infection at weaning and significantly reduced the amount of viral shedding. This study clearly showed that the viral load in serum is negatively correlated with the number of PRRSV-specific IFN-γ secreting cells in PBMC. This suggests that the T-cell driven immune response in newborn piglets is already highly effective.

## Figures and Tables

**Table 1:** Least squares mean (LSM) differences of the five investigated histologic lung lesions between the two treatment groups. The scores for each lesion were ranked and the ranks were analyzed with a general linear mixed model with fixed effect: treatment and random effect: block.

Histologic Lesion	LSM Difference between T01 and T02	Degrees of Freedom	T-Value	*p*-Value
Intra-alveolar accumulation of inflammatory cells	18.4	38	8.23	<0.0001
Perivascular accumulation of inflammatory cells	17.4	36	7.35	<0.0001
Necrotic debris	17.5	38	7.29	<0.0001
Pneumocytic hypertrophy and hyperplasia	17.2	38	6.98	<0.0001
Septal infiltration with mononuclear cells	16.0	38	5.98	<0.0001

**Table 2:** Least squares mean (LSM) differences of the log_10_ transformed viremia and nasal shedding data between the two treatment groups per study day. The data was analyzed using a general linear repeated measures mixed model with fixed effects: treatment, time point, and treatment and time point interaction and random effects: block and piglet within block by treatment.

Study Day	Sample	LSM Difference between T01 and T02	Standard Error	Degrees of Freedom	*p*-Value
D31	Serum	3.40	0.40	56	<0.0001
	Nasal swab	0.54	0.10	42	<0.0001
D33	Serum	3.47	0.40	56	<0.0001
	Nasal swab	0.72	0.13	41	<0.0001
D35	Serum	2.88	0.40	56	<0.0001
	Nasal swab	0.08	0.10	37	0.405
D38	Serum	2.91	0.40	56	<0.0001
	Nasal swab	0.05	0.04	41	0.157
D41/42	Serum	3.04	0.40	56	<0.0001
	Nasal swab	0.59	0.17	36	0.002

**Table 3:** Least squares mean (LSM) differences of IFN-γ ELISpot results between the two treatment groups per study day. The data was analyzed using a general linear repeated measures mixed model with fixed effects: treatment, time point, and treatment and time point interaction. Random effects were block and piglet within block by treatment.

Study Day	LSM Difference between T01 and T02	Standard Error	Degrees of Freedom	*p*-Value
D28	−3.5	0.2	24	<0.0001
D35	−2.7	0.3	34	<0.0001
D41/42	−2.0	0.4	36	<0.0001

**Table 4:** Least squares mean (LSM) differences of ELISA results between the two treatment groups per study day. The data was analyzed using a general linear repeated measures mixed model with fixed effects: treatment, time point, and treatment and time point interaction. Random effects were block and piglet within block by treatment.

Study Day	LSM Difference between T01 and T02	Standard Error	Degrees of Freedom	*p*-Value
D28	−2.4	0.2	74	<0.0001
D41/42	−0.7	0.2	74	0.002

## Data Availability

The data presented in this study are available on request from the corresponding author. The data are not publicly available due to privacy reasons by the funder.

## References

[1] Holtkamp D.J., Kliebenstein J.B., Neumann E.J., Zimmerman J.J., Rotto H.F., Yoder T.K., Wang C., Yeske P.E., Mowrer C.L., Haley C.A. (2013). Assessment of the economic impact of porcine reproductive and respiratory syndrome virus on United States pork producers. J. Swine Health Prod..

[2] Oppeneder A., Griessler A., Voglmayr T., Reitböck R., Renzhammer R., Ritzmann M., Stadler J., Ladinig A. (2020). Economic impact of a PRRS virus introduction via semen into farms with different PRRSV status. Berl. Münchener Tierärztliche Wochenschr..

[3] Kuhn J.H., Lauck M., Bailey A.L., Shchetinin A.M., Vishnevskaya T.V., Bao Y.M., Ng T.F.F., LeBreton M., Schneider B.S., Gillis A. (2016). Reorganization and expansion of the nidoviral family Arteriviridae. Arch. Virol..

[4] Shi M., Lam T.T., Hon C.C., Murtaugh M.P., Davies P.R., Hui R.K., Li J., Wong L.T., Yip C.W., Jiang J.W. (2010). Phylogeny-based evolutionary, demographical, and geographical dissection of North American type 2 porcine reproductive and respiratory syndrome viruses. J. Virol..

[5] Stadejek T., Stankevicius A., Murtaugh M.P., Oleksiewicz M.B. (2013). Molecular evolution of PRRSV in Europe: Current state of play. Vet. Microbiol..

[6] Balka G., Podgorska K., Brar M.S., Balint A., Cadar D., Celer V., Denes L., Dirbakova Z., Jedryczko A., Marton L. (2018). Genetic diversity of PRRSV 1 in Central Eastern Europe in 1994-2014: Origin and evolution of the virus in the region. Sci. Rep..

[7] Balint A., Balka G., Horvath P., Kecskemeti S., Dan A., Farsang A., Szeredi L., Banyai K., Bartha D., Olasz F. (2015). Full-length genome sequence analysis of a Hungarian porcine reproductive and respiratory syndrome virus isolated from a pig with severe respiratory disease. Arch. Virol..

[8] Frydas I.S., Trus I., Kvisgaard L.K., Bonckaert C., Reddy V.R., Li Y., Larsen L.E., Nauwynck H.J. (2015). Different clinical, virological, serological and tissue tropism outcomes of two new and one old Belgian type 1 subtype 1 porcine reproductive and respiratory virus (PRRSV) isolates. Vet. Res..

[9] Sinn L.J., Klingler E., Lamp B., Brunthaler R., Weissenböck H., Rümenapf T., Ladinig A. (2016). Emergence of a virulent porcine reproductive and respiratory syndrome virus (PRRSV) 1 strain in Lower Austria. Porcine Health Manag..

[10] Canelli E., Catella A., Borghetti P., Ferrari L., Ogno G., De Angelis E., Corradi A., Passeri B., Bertani V., Sandri G. (2017). Phenotypic characterization of a highly pathogenic Italian porcine reproductive and respiratory syndrome virus (PRRSV) type 1 subtype 1 isolate in experimentally infected pigs. Vet. Microbiol..

[11] Dürlinger S., Balka G., Rathkjen P.H., Kraft C., Morgenstern R., Rümenapf T., Ladinig A. Efficacy of Ingelvac PRRSFLEX® EU against experimental challenge with PRRSV AUT15-33 (“ACRO” PRRSV). Proceedings of the 11th European Symposium of Porcine Health Management (ESPHM).

[12] Nathues H., Alarcon P., Rushton J., Jolie R., Fiebig K., Jimenez M., Geurts V., Nathues C. (2017). Cost of porcine reproductive and respiratory syndrome virus at individual farm level—An economic disease model. Prev. Vet. Med..

[13] Bonckaert C., van der Meulen K., Rodriguez-Ballara I., Pedrazuela Sanz R., Martinez M.F., Nauwynck H.J. (2016). Modified-live PRRSV subtype 1 vaccine UNISTRAIN((R)) PRRS provides a partial clinical and virological protection upon challenge with East European subtype 3 PRRSV strain Lena. Porcine Health Manag..

[14] Canelli E., Catella A., Borghetti P., Ferrari L., Ogno G., De Angelis E., Bonilauri P., Guazzetti S., Nardini R., Martelli P. (2018). Efficacy of a modified-live virus vaccine in pigs experimentally infected with a highly pathogenic porcine reproductive and respiratory syndrome virus type 1 (HP-PRRSV-1). Vet. Microbiol..

[15] Thomann B., Rushton J., Schuepbach-Regula G., Nathues H. (2020). Modeling Economic Effects of Vaccination Against Porcine Reproductive and Respiratory Syndrome: Impact of Vaccination Effectiveness, Vaccine Price, and Vaccination Coverage. Front. Vet. Sci..

[16] Charerntantanakul W. (2012). Porcine reproductive and respiratory syndrome virus vaccines: Immunogenicity, efficacy and safety aspects. World J. Virol..

[17] Balasch M., Fort M., Taylor L.P., Calvert J.G. (2018). Vaccination of 1-day-old pigs with a porcine reproductive and respiratory syndrome virus (PRRSV) modified live attenuated virus vaccine is able to overcome maternal immunity. Porcine Health Manag..

[18] Jeong J., Kim S., Park K.H., Kang I., Park S.J., Yang S., Oh T., Chae C. (2018). Vaccination with a porcine reproductive and respiratory syndrome virus vaccine at 1-day-old improved growth performance of piglets under field conditions. Vet. Microbiol..

[19] Balasch M., Fort M., Taylor L.P., Diaz I., Mateu E., Calvert J.G. (2019). Immune response development after vaccination of 1-day-old naive pigs with a Porcine Reproductive and Respiratory Syndrome 1-based modified live virus vaccine. Porcine Health Manag..

[20] Mateu E., Diaz I. (2008). The challenge of PRRS immunology. Vet. J..

[21] Loving C.L., Osorio F.A., Murtaugh M.P., Zuckermann F.A. (2015). Innate and adaptive immunity against Porcine Reproductive and Respiratory Syndrome Virus. Vet. Immunol. Immunopathol..

[22] Holtkamp D.J., Polson D.D., Torremorell M., Morrison B., Classen D.M., Becton L., Henry S., Rodibaugh M.T., Rowland R.R., Snelson H. (2011). Terminology for classifying swine herds by porcine reproductive and respiratory syndrome virus status. J. Swine Health Prod..

[23] Stevenson G.W., Van Alstine W.G., Kanitz C.L., Keffaber K.K. (1993). Endemic porcine reproductive and respiratory syndrome virus infection of nursery pigs in two swine herds without current reproductive failure. J. Vet. Diagn. Investig..

[24] De Regge N., Cay B. (2016). Comparison of PRRSV Nucleic Acid and Antibody Detection in Pen-Based Oral Fluid and Individual Serum Samples in Three Different Age Categories of Post-Weaning Pigs from Endemically Infected Farms. PLoS ONE.

[25] Lopez W.A., Angulo J., Zimmerman J.J., Linhares D.C.L. (2018). Porcine reproductive and respiratory syndrome monitoring in breeding herds using processing fluids. J. Swine Health Prod..

[26] Corzo C.A., Mondaca E., Wayne S., Torremorell M., Dee S., Davies P., Morrison R.B. (2010). Control and elimination of porcine reproductive and respiratory syndrome virus. Virus Res..

[27] Johnson W., Roof M., Vaughn E., Christopher-Hennings J., Johnson C.R., Murtaugh M.P. (2004). Pathogenic and humoral immune responses to porcine reproductive and respiratory syndrome virus (PRRSV) are related to viral load in acute infection. Vet. Immunol. Immunopathol..

[28] Martelli P., Gozio S., Ferrari L., Rosina S., De Angelis E., Quintavalla C., Bottarelli E., Borghetti P. (2009). Efficacy of a modified live porcine reproductive and respiratory syndrome virus (PRRSV) vaccine in pigs naturally exposed to a heterologous European (Italian cluster) field strain: Clinical protection and cell-mediated immunity. Vaccine.

[29] Murtaugh M.P., Genzow M. (2011). Immunological solutions for treatment and prevention of porcine reproductive and respiratory syndrome (PRRS). Vaccine.

[30] Li X., Galliher-Beckley A., Pappan L., Trible B., Kerrigan M., Beck A., Hesse R., Blecha F., Nietfeld J.C., Rowland R.R. (2014). Comparison of host immune responses to homologous and heterologous type II porcine reproductive and respiratory syndrome virus (PRRSV) challenge in vaccinated and unvaccinated pigs. Biomed. Res. Int.

[31] Trus I., Bonckaert C., van der Meulen K., Nauwynck H.J. (2014). Efficacy of an attenuated European subtype 1 porcine reproductive and respiratory syndrome virus (PRRSV) vaccine in pigs upon challenge with the East European subtype 3 PRRSV strain Lena. Vaccine.

[32] Wensvoort G., Terpstra C., Pol J.M., ter Laak E.A., Bloemraad M., de Kluyver E.P., Kragten C., van Buiten L., den Besten A., Wagenaar F. (1991). Mystery swine disease in The Netherlands: The isolation of Lelystad virus. Vet. Q..

[33] Rossow K.D., Bautista E.M., Goyal S.M., Molitor T.W., Murtaugh M.P., Morrison R.B., Benfield D.A., Collins J.E. (1994). Experimental porcine reproductive and respiratory syndrome virus infection in one-, four-, and 10-week-old pigs. J. Vet. Diagn. Investig..

[34] Lunney J.K., Fang Y., Ladinig A., Chen N., Li Y., Rowland B., Renukaradhya G.J. (2016). Porcine Reproductive and Respiratory Syndrome Virus (PRRSV): Pathogenesis and Interaction with the Immune System. Annu. Rev. Anim. Biosci..

[35] Balka G., Ladinig A., Ritzmann M., Saalmüller A., Gerner W., Käser T., Jakab C., Rusvai M., Weissenböck H. (2013). Immunohistochemical characterization of type II pneumocyte proliferation after challenge with type I porcine reproductive and respiratory syndrome virus. J. Comp. Pathol..

[36] Duan X., Nauwynck H.J., Pensaert M.B. (1997). Virus quantification and identification of cellular targets in the lungs and lymphoid tissues of pigs at different time intervals after inoculation with porcine reproductive and respiratory syndrome virus (PRRSV). Vet. Microbiol..

[37] Labarque G., Van Gucht S., Nauwynck H., Van Reeth K., Pensaert M. (2003). Apoptosis in the lungs of pigs infected with porcine reproductive and respiratory syndrome virus and associations with the production of apoptogenic cytokines. Vet. Res..

[38] Lee S.M., Kleiboeker S.B. (2007). Porcine reproductive and respiratory syndrome virus induces apoptosis through a mitochondria-mediated pathway. Virology.

[39] Zimmerman J.J., Dee S.A., Holtkamp D.J., Murtaugh M.P., Stadejek T., Stevenson G.W., Torremorell M., Yang H., Zhang J., Zimmerman J.J., Karriker L.A., Ramirez A., Schwartz K.J., Stevenson G.W., Zhang J. (2019). Porcine Reproductive and Respiratory Syndrome Viruses (Porcine Arteriviruses). Diseases of Swine.

[40] Drigo M., Franzo G., Tucciarone C.M., Manfredda A., Zanardelli P., Basano F.S., Nazzari R. Effect of early PRRSV (Porcine Reproductive and Respiratory Syndrome Virus) vaccination on pig health and performance: The earlier the better?. Proceedings of the 11th European Symposium of Porcine Health Management (ESPHM).

[41] Wills R.W., Zimmerman J.J., Yoon K.J., Swenson S.L., Hoffman L.J., McGinley M.J., Hill H.T., Platt K.B. (1997). Porcine reproductive and respiratory syndrome virus: Routes of excretion. Vet. Microbiol..

[42] Cho J.G., Dee S.A., Deen J., Trincado C., Fano E., Jiang Y., Faaberg K., Murtaugh M.P., Guedes A., Collins J.E. (2006). The impact of animal age, bacterial coinfection, and isolate pathogenicity on the shedding of porcine reproductive and respiratory syndrome virus in aerosols from experimentally infected pigs. Can. J. Vet. Res..

[43] Pileri E., Gibert E., Soldevila F., Garcia-Saenz A., Pujols J., Diaz I., Darwich L., Casal J., Martin M., Mateu E. (2015). Vaccination with a genotype 1 modified live vaccine against porcine reproductive and respiratory syndrome virus significantly reduces viremia, viral shedding and transmission of the virus in a quasi-natural experimental model. Vet. Microbiol..

[44] Yoon I.J., Joo H.S., Goyal S.M., Molitor T.W. (1994). A modified serum neutralization test for the detection of antibody to porcine reproductive and respiratory syndrome virus in swine sera. J. Vet. Diagn. Investig..

[45] Lopez O.J., Osorio F.A. (2004). Role of neutralizing antibodies in PRRSV protective immunity. Vet. Immunol. Immunopathol..

[46] Cancel-Tirado S.M., Evans R.B., Yoon K.J. (2004). Monoclonal antibody analysis of porcine reproductive and respiratory syndrome virus epitopes associated with antibody-dependent enhancement and neutralization of virus infection. Vet. Immunol. Immunopathol..

[47] Fablet C., Renson P., Eono F., Mahe S., Eveno E., Le Dimna M., Normand V., Lebret A., Rose N., Bourry O. (2016). Maternally-derived antibodies (MDAs) impair piglets’ humoral and cellular immune responses to vaccination against porcine reproductive and respiratory syndrome (PRRS). Vet. Microbiol..

[48] Renson P., Fablet C., Andraud M., Normand V., Lebret A., Paboeuf F., Rose N., Bourry O. (2019). Maternally-derived neutralizing antibodies reduce vaccine efficacy against porcine reproductive and respiratory syndrome virus infection. Vaccine.

[49] Talker S.C., Käser T., Reutner K., Sedlak C., Mair K.H., Koinig H., Graage R., Viehmann M., Klingler E., Ladinig A. (2013). Phenotypic maturation of porcine NK- and T-cell subsets. Dev. Comp. Immunol..

[50] Chase C., Lunney J.K., Zimmerman J.J., Karriker L.A., Ramirez A., Schwartz K.J., Stevenson G.W., Zhang J. (2019). Immune System. Diseases of Swine.

[51] Diaz I., Darwich L., Pappaterra G., Pujols J., Mateu E. (2005). Immune responses of pigs after experimental infection with a European strain of Porcine reproductive and respiratory syndrome virus. J. Gen. Virol..

[52] Mair K.H., Koinig H., Gerner W., Hohne A., Bretthauer J., Kroll J.J., Roof M.B., Saalmüller A., Stadler K., Libanova R. (2015). Carbopol improves the early cellular immune responses induced by the modified-life vaccine Ingelvac PRRS(R) MLV. Vet. Microbiol..

[53] Rowland R.R., Robinson B., Stefanick J., Kim T.S., Guanghua L., Lawson S.R., Benfield D.A. (2001). Inhibition of porcine reproductive and respiratory syndrome virus by interferon-gamma and recovery of virus replication with 2-aminopurine. Arch. Virol..

[54] Zuckermann F.A., Garcia E.A., Luque I.D., Christopher-Hennings J., Doster A., Brito M., Osorio F. (2007). Assessment of the efficacy of commercial porcine reproductive and respiratory syndrome virus (PRRSV) vaccines based on measurement of serologic response, frequency of gamma-IFN-producing cells and virological parameters of protection upon challenge. Vet. Microbiol..

[55] Straw B.E., Backstrom L., Leman A.D. (1986). Examination of Swine at Slaughter.2. Findings at Slaughter and Their Significance. Comp. Cont. Educ. Pract..

